# The relationship between dietary inflammatory index and osteoporosis among chronic kidney disease population

**DOI:** 10.1038/s41598-023-49824-5

**Published:** 2023-12-18

**Authors:** Xinxuan Meng, Wenxin Sha, Xiaowei Lou, Jianghua Chen

**Affiliations:** 1 Kidney Disease Center, First Affiliated Hospital, College of Medicine, Zhejiang University, Hangzhou, 310000 China; 2 College of Medicine, Zhejiang University, Hangzhou, China; 3 Key Laboratory of Kidney Disease Prevention and Control Technology, Zhejiang Province, Hangzhou, China; 4 National Key Clinical Department of Kidney Diseases, Hangzhou, China; 5 Institute of Nephrology，Zhejiang University, Hangzhou, China; 6 Zhejiang Clinical Research Center of Kidney and Urinary System Disease, Hangzhou, China

**Keywords:** Nephrology, Risk factors

## Abstract

Dietary inflammation index (DII) is an epidemiological survey tool to evaluate dietary inflammation potential. Osteoporosis, whose development is deeply affected by inflammation, may be also affected by dietary inflammatory patterns. However, the relationship between DII and osteoporosis is unclear for chronic kidney disease (CKD) population. Our study involved 526 CKD patients from the US National Health and Nutrition Examination Survey (NHANES). DII levels were stratified into four quantile groups. Multivariable regression models were used to examine the association between DII and osteoporosis. Restricted cubic splines and subgroup analysis were additionally adopted. Results showed that the overall prevalence of osteoporosis among CKD patients was 25.3%. After fully adjusted, OR (95% confidence interval) for Q4 group compared with Q3 (reference group) in total and female population were 2.09 (1.05, 4.23) and 2.80 (1.14, 7.08), respectively. Subgroup analysis indicated that these results had no interaction with age, gender, body mass index (BMI), renal function, urinary protein, calcium, phosphorus and total 25-hydroxyvitamin D. DII was negatively correlated with lumbar spine bone mineral density (BMD) in CKD population (*P* < 0.05). Therefore, in CKD patients, higher DII was associated with higher osteoporosis risk and lower BMD of lumber spine, especially in female. Anti-inflammatory diet patterns may be a protective intervention for some CKD-related osteoporosis.

## Introduction

Chronic kidney disease (CKD) is a global public health problem, with increasing worldwide prevalence to an estimation of 840 million individuals in 2017^1^ and adverse outcomes causing 1.2 million deaths and 28 million years of life lost each year^2,3^. Mineral and bone metabolism imbalances are common in CKD population and are a significant contributor to increased morbidity and decreased quality of life^4^. This metabolic disorder, also referred as chronic kidney disease-mineral bone disorder (CKD-MBD), directly exposes patients with CKD to a higher fracture risk because of deteriorated bone quality and quantity^5^. Parathormone (PTH), calcitriol or 1, 25(OH)_2_D_3_ (the natural most active form of the hormonal system of vitamin D), calcidiol or 25(OH)D_3_ (substrate of calcitriol), calcitonin and fibroblast growth factor 23 (FGF23)/klotho are the classical key calciotropic hormones regulating calcium and phosphate in CKD patients^6^. Recently, a number of studies are unveiling the association between bone health with the immune system, opening up a new field nicknamed osteoimmunology (detailed in^7^). This inspires further studies demonstrating that inflammation and system immunity can affect bone metabolism in CKD patients since these patients tend to be in a state of chronic inflammation. For example, T-helper 17 (Th17) cells, which play a pivotal role in inducing and sustaining the bone lesions through IL-17 secretion, can induce the inflammatory reaction, incremental receptor activator of nuclear factor kappa-B ligand (RANKL) expression on mesenchymal cells, osteoclast recruitment, and bone resorption and destruction^8^. Other cytokines such as interferon-γ, IL-4, and IL-10, produced by other subsets of Th cells, together with the osteoprotegerin (OPG) produced by B cells, also participate in the inhibition of osteoclast formation and differentiation^9^. These evidence inspires further studies demonstrating that inflammation and system immunity can affect bone metabolism in CKD patients since they tend to be in a state of chronic inflammation. Recently, a study found that the cytokine increment did interact with osteoblasts and osteoclasts, affecting bone remodeling and the extent of bone erosion during inflammatory responses in CKD patients^10^.

Existing studies have pointed out that dietary pattern plays an important role in developing chronic inflammation^11,12^. As mentioned above, inflammation is closely related to osteoporosis in CKD patients^13^. The dietary inflammatory index (DII), first proposed by Shivappa et al.^14^ after extensive literature search, is a scoring system to quantify the inflammatory effects of nutrients and foods. It is designed by assigning a score for each of 45 food parameters involved in regulating the levels of 6 specific inflammatory biomarkers (IL-1β, IL-4, IL6, IL-10, TNF-α, and C-reactive protein)^15^. We used to lay more emphasis on the content of nutrients in the diet to evaluate the effects of diet on CKD patients^16^. Here, DII offers us a brand new perspective in evaluating the unwanted inflammatory effects brought by various nutrients and food.

The National Health and Nutrition Examination Survey (NHANES), conducted by the Center for Disease Control and Prevention (CDC), has proven to be a feasible tool to obtain DII^17–19^. A previous study has examined the relationship between DII and osteoporosis based on the NHANES^19^. However, their results were only partly consistent with another study where diet with high inflammatory potential was significantly associated with an increased risk of osteoporosis in female, but not in male^12^. Few researchers have focused on the CKD population and the existing results showed inconsistency of the relationship between DII and osteoporosis in different studies, so the present cross-sectional study aimed to explore the association between DII and osteoporosis in patients with CKD by using the NHANES data.

## Results

### Characteristics of study participants

**Figure 1:**
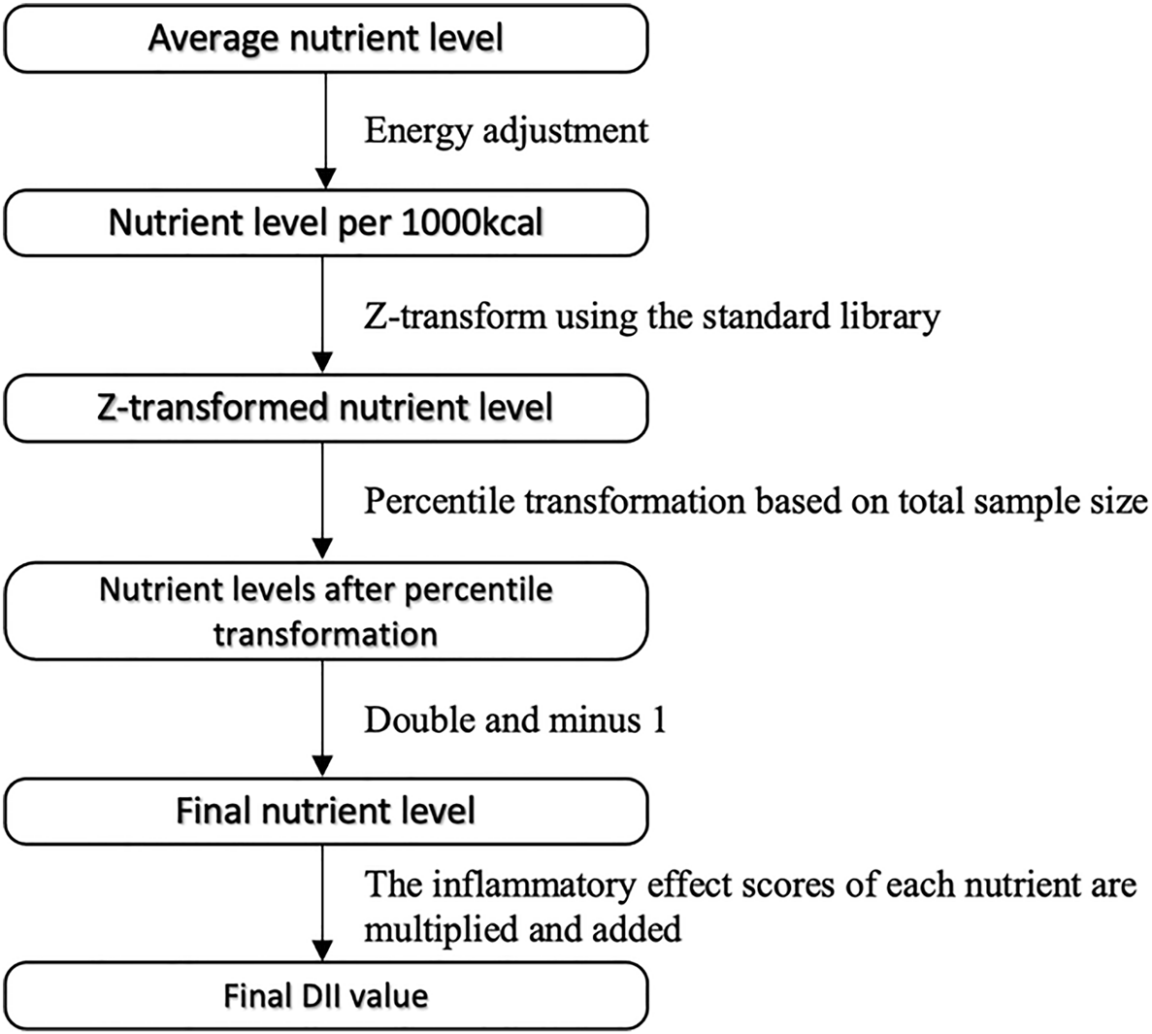
Calculation process of DII.

There were 526 participants (43.5% male) from 2 NHANES cycles in this study (Fig. 1, refer to Figure S1 for the sample size calculation). The characteristics of participants are presented at the overall and quantile DII levels in Table 1. The average age of participants was 63.25 years. A total of 133 (25.3%) among the patients developed osteoporosis. The overall DII score ranged from – 5.41 to 4.23. According to the DII score range, we divided the subjects into four groups on quantiles: quantile 1 (Q1, DII − 5.41 to − 1.22, n = 131), quantile 2 (Q2, DII − 1.22 to − 0.02, n = 132), quantile 3 (Q3, DII − 0.02 to 1.21, n = 131), and quantile 4 (Q4, DII 1.21 ~ 4.23, n = 132). Significant differences in age (*P* < 0.001), gender (*P* = 0.003), smoking and drinking habits (*P* = 0.005, and 0.006, respectively), and bone mineral density (BMD) at the total hip (*P* = 0.048) were observed among quantile groups but not in the prevalence of osteoporosis. In laboratory tests, the levels of serum albumin (*P* = 0.014), serum calcium (*P* = 0.050), and serum total 25-hydroxyvitamin D (*P* < 0.001) were significantly different among the quartile groups.

**Table 1:** Characteristics of study participants according to DII.

Variables	Overall	DII				*P* value
	(N = 526)	Q1 (N = 131)	Q2 (N = 132)	Q3 (N = 131)	Q4 (N = 132)	
DII, min ~ max	− 5.41 ~ 4.23	− 5.41 ~ − 1.22	− 1.22 ~ − 0.02	− 0.02 ~ 1.21	1.21 ~ 4.23	
Male (n, %)	229 (43.5%)	47 (35.9%)^a^	67 (50.8%)	46 (35.1%)^b^	69 (52.3%)^ab^	0.003**
Age, y	63.25 ± 11.33	66.07 ± 10.57^ab^	64.55 ± 11.03^c^	62.41 ± 11.56^a^	60.01 ± 11.33^bc^	< 0.001**
Race (n, %)						0.197
Mexican American	54 (10.3%)	10 (7.6%)	17 (12.9%)	19 (14.5%)	8 (6.1%)	
Other Hispanic	40 (7.6%)	11 (8.4%)	13 (9.8%)	9 (6.9%)	7 (5.3%)	
Non-Hispanic White	208 (39.5%)	52 (39.7%)	47 (35.6%)	52 (39.7%)	57 (43.2%)	
Non-Hispanic Black	156 (29.7%)	33 (25.2%)	33 (25.0%)	40 (30.5%)	50 (37.9%)	
Other Race	68 (12.9%)	25 (19.1%)	22 (16.7%)	11 (8.4%)	10 (7.6%)	
Income (n, %)						0.500
Not poor	106 (20.2%)	27 (20.6%)	22 (16.7%)	25 (19.1%)	32 (24.2%)	
Near poor	233 (44.3%)	49 (37.4%)	59 (44.7%)	64 (48.9%)	61 (46.2%)	
Poor	187 (35.6%)	55 (42.0%)	51 (38.6%)	42 (32.1%)	39 (29.5%)	
BMI, kg/m^2^						0.433
< 25.0	102 (19.5%)	24 (18.5%)	28 (21.3%)	26 (20.0%)	24 (18.4%)	
25.0 ~ 30	146 (27.9%)	45 (34.6%)	37 (28.0%)	31 (23.8%)	33 (25.2%)	
≥ 30	275 (52.6%)	61 (46.9%)	67 (50.8%)	73 (56.2%)	74 (56.5%)	
Smoking (n, %)	245 (46.6%)	44 (33.6%)^ab^	70 (53.0%)^a^	62 (47.3%)	69 (52.3%)^b^	0.005**
Drinking (n, %)	153 (51.0%)	22 (36.1%)^ab^	35 (45.5%)	44 (54.3%)^a^	52 (64.2%)^b^	0.006**
Hypertension (n, %)	340 (64.9%)	84 (64.1%)	85 (64.9%)	79 (60.8%)	92 (69.7%)	0.503
Diabetes (n, %)	162 (30.9%)	41 (31.3%)	45 (34.1%)	33 (25.2%)	43 (32.8%)	0.406
Cardiovascular disease (n, %)	48 (9.1%)	8 (6.1%)	12 (9.1%)	16 (12.2%)	12 (9.1%)	0.389
Malignant tumor (n, %)	83 (15.8%)	21 (16.0%)	18 (13.6%)	24 (18.3%)	20 (15.2%)	0.768
Stroke (n, %)	46(8.7%)	10 (7.6%)	6 (4.5%)	15 (11.5%)	15 (11.4%)	0.141
eGFR, ml/min	61.11 [53.39, 84.69]	60.59 [52.94, 77.65]	65.26 [54.04, 87.92]	60.16 [53.03, 91.03]	61.34 [54.50, 82.66]	0.714
ACR, mg/g						0.890
A1	179 (34.0%)	47 (35.9%)	43 (32.6%)	50 (38.2%)	39 (29.5%)	
A2	286 (54.4%)	70 (53.4%)	69 (52.3%)	68 (51.9%)	79 (59.8%)	
A3	61 (11.6%)	14 (10.7%)	20 (15.2%)	13 (9.9%)	14 (10.6%)	
Hemoglobin, g/L	13.59 ± 1.62	13.46 ± 1.72	13.75 ± 1.50	13.41 ± 1.39	13.75 ± 1.82	0.171
NLR	2.06 [1.50, 2.82]	1.95 [1.46, 2.53]	2.18 [1.58, 2.82]	2.00 [1.54, 2.71]	2.18 [1.41, 3.09]	0.442
Albumin, g/L	40.89 ± 3.32	41.64 ± 3.09^ab^	40.56 ± 3.77^a^	40.93 ± 2.99	40.43 ± 3.26^b^	0.014*
Calcium, mmol/L	2.36 ± 0.10	2.38 ± 0.12^a^	2.35 ± 0.09	2.35 ± 0.09^a^	2.36 ± 0.10	0.050*
Phosphorus, mmol/L	1.20 ± 0.19	1.22 ± 0.18	1.21 ± 0.20	1.18 ± 0.18	1.19 ± 0.19	0.418
Uric acid, μmol/L	339.00 [285.50, 413.40]	339.00 [279.60, 395.55]	336.05 [284.02, 404.50]	339.00 [291.50, 410.40]	356.90 [282.55, 428.30]	0.566
Total 25-hydroxyvitamin D, mmol/L	71.35 [50.12, 97.90]	83.70 [61.95, 105.50]^abc^	70.70 [47.77, 94.67]^a^	69.90 [51.45, 97.25]^b^	59.30 [41.38, 86.88]^c^	< 0.001**
Total hip BMD, g/cm^2^	0.92 ± 0.17	0.89 ± 0.15^a^	0.93 ± 0.17	0.92 ± 0.18	0.94 ± 0.16^a^	0.048*
Femoral neck BMD, g/cm^2^	0.76 ± 0.15	0.74 ± 0.14	0.76 ± 0.16	0.76 ± 0.15	0.78 ± 0.14	0.153
Lumbar spine BMD, g/cm^2^	1.01 ± 0.18	0.99 ± 0.16	1.02 ± 0.18	1.02 ± 0.20	1.03 ± 0.18	0.186
Osteoporosis (n, %)	133 (25.3%)	41 (31.3%)	31 (23.5%)	30 (22.9%)	31 (23.5%)	0.356
Calcium intake, mg/d	688.50 [460.50, 1073.75]	706.00 [502.00, 1123.00]	678.00 [476.75, 1048.25]	674.00 [426.50, 1112.00]	726.50 [429.75, 1050.75]	0.828
Phosphorus intake, mg/d	1137.00 [794.50, 1578.00]	1091.00 [795.50, 1547.50]	1173.50 [846.50, 1607.00]	1079.00 [788.00, 1547.00]	1172.00 [751.00, 1569.25]	0.764
Steroids use (n, %)	44 (8.4%)	7 (5.3%)	11 (8.4%)	10 (7.7%)	16 (12.2%)	0.262
Estrogen (n,%)	80(15.2%)	23(17.6%)	17(12.9%)	23(17.6%)	17(12.9%)	0.526
Anti-osteoporosis drugs (n,%)	38(7.2%)	13 (9.9%)	12(9.1%)	7(5.3%)	6(4.5%)	0.239
Physical activity (n, %)	141(26.8%)	35 (26.7%)	34 (25.8%)	32 (24.4%)	40 (30.3%)	0.735

*DII* Dietary inflammatory index; *BMI* Body mass index; *eGFR* Estimated glomerular filtration rate; *NLR* Neutrophil to lymphocyte ratio; *ACR* Albumin to creatinine ratio (A1, ACR < 30 mg/g; A2, ACR 30–300 mg/g; A3, ACR > 300 mg/g); *BMD* Bone mineral density; Total 25-Hydroxyvitamin D: the sum of 25-hydroxyvitamin D2 and 25-hydroxyvitamin D3, but excludes epi-25-hydroxyvitamin D3; ^a, b, c^statistically significance between 2 groups using the Bonferroni method; *p < 0.05; **p < 0.01.

### Association between DII and the risk of osteoporosis

The results of the adjusted odds ratios (aORs) for risk of osteoporosis according to the quantile groups of DII scores are shown in Table 2. In total CKD population, the significantly increased aORs (95% CI) indicating a higher risk of osteoporosis in Q4 group compared with Q3 across model 2 and model 3 were 2.35 (1.25, 4.41), and 2.09 (1.05, 4.23), respectively. Notably, the risk of osteoporosis in the Q1 group was also significantly higher than that in the Q3 group in model 3 (aOR 1.96, 95% CI 1.01 to 3.85). The results in the female CKD population were generally consistent with those in the general CKD population with aORs in Q4 group across all three models gradually increased as more covariates being corrected (all *P* < 0.05). However, no association between DII and osteoporosis was observed in men across three models (*P* > 0.05).

**Table 2:** Association of DII and osteoporosis.

Models	DII groups	OR[95% CI]		
		All group	Male group	Female group
Model 1	Q1	1.46 (0.82, 2.60)	1.23 (0.35, 4.26)	1.55 (0.79, 3.02)
	Q2	1.32 (0.64, 2.73)	1.37 (0.31, 6.04)	1.66 (0.74, 3.73)
	**Q3**	**Reference**	**Reference**	**Reference**
	Q4	1.13 (0.59, 2.14)	0.75 (0.21, 2.66)	2.15 (1.04, 4.46)*
Model 2	Q1	1.44 (0.75, 2.77)	0.98 (0.26, 3.63)	1.71 (0.79, 3.71)
	Q2	1.72 (0.80, 3.72)	1.39 (0.30, 6.34)	1.87 (0.73, 4.77)
	**Q3**	**Reference**	**Reference**	**Reference**
	Q4	2.35 (1.25, 4.41)*	0.97 (0.24, 4.00)	3.69 (1.74, 7.84)*
Model 3	Q1	1.96 (1.01, 3.85)*	0.41 (0.09, 1.64)	3.76 (1.62, 9.12)*
	Q2	1.25 (0.63, 2.50)	0.57 (0.16, 2.07)	1.59 (0.67, 3.83)
	**Q3**	**Reference**	**Reference**	**Reference**
	Q4	2.09 (1.05, 4.23)*	1.21 (0.32,4.85)	2.80 (1.14, 7.08)*

Model 1: Not adjusted for any other variables.

Model 2: Adjusted for age, gender and race.

Model 3: Adjusted for age, gender, race, BMI group, smoking, steroids use, calcium intake, phosphorus intake, serum calcium, serum phosphorus, total 25-hydroxyvitamin D, albumin, estrogen, anti-osteoporosis drugs, and physical activity.

*OR* Odds ratio; *CI* Confidence interval; *p < 0.05.

**Figure 2:**
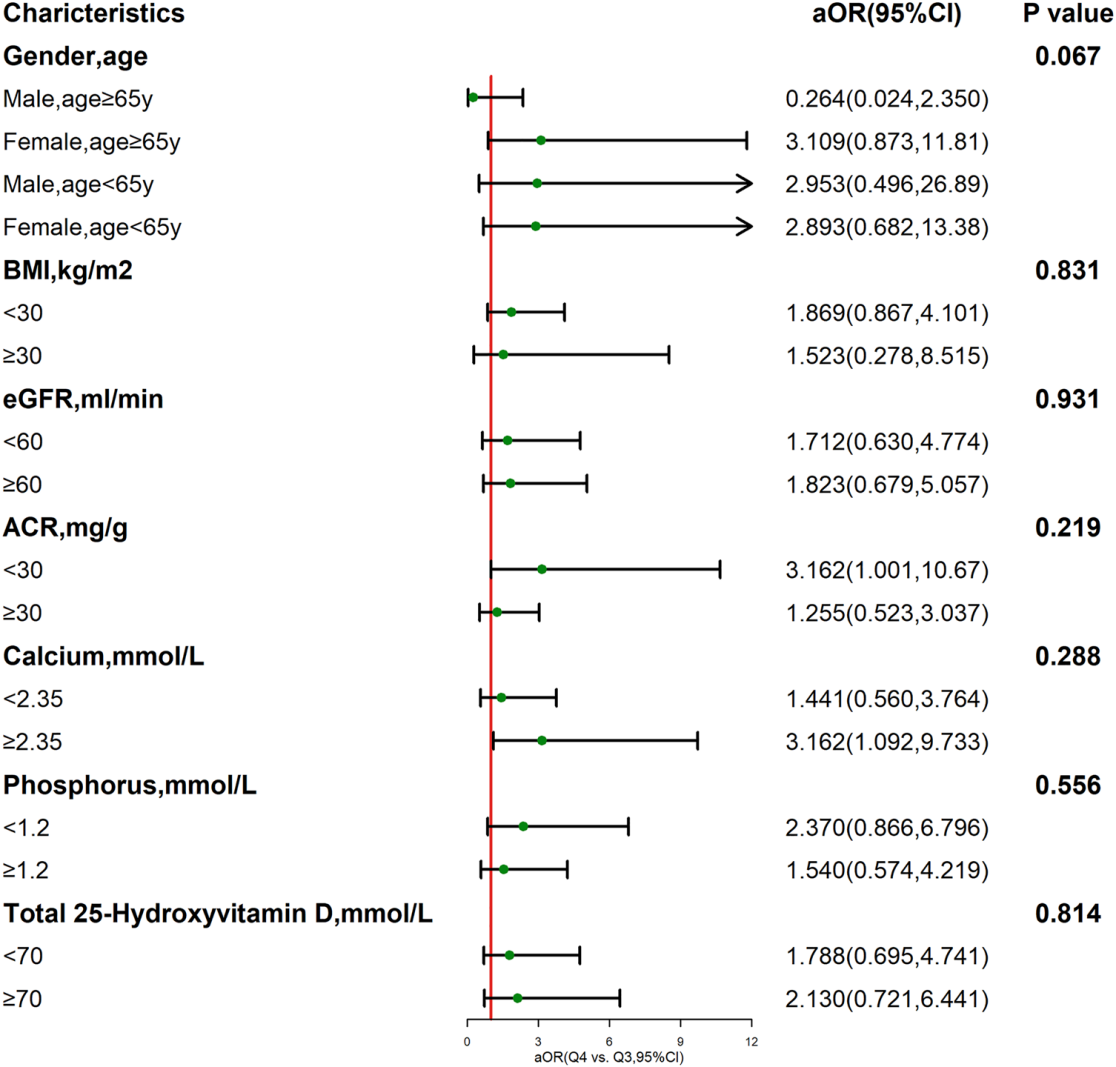
Association between DII and osteoporosis risk in CKD population stratified by different factors. The forest plot shows ORs and 95% CIs for developing osteoporosis in CKD population stratified by gender, age, BMI, eGFR, ACR, serum calcium, serum phosphorus and total 25-hydroxyvitamin D. The models used were adjusted for age, gender, race, BMI group, smoking, steroids use, calcium intake, phosphorus intake, serum calcium, serum phosphorus, total 25-hydroxyvitamin D, albumin, estrogen, anti-osteoporosis drugs, and physical activity. Serum calcium, serum phosphorus and total 25-hydroxyvitamin D in this figure were stratified by median of whole group.

Subgroups analysis comparing between Q3 group and Q4 group showed that the effect of DII on osteoporosis was not affected by age, gender, body mass index (BMI), renal function, urinary protein, serum calcium, phosphorous, total 25-hydroxyvitamin D (all *P* > 0.05, see Fig. 2).

**Figure 3:**
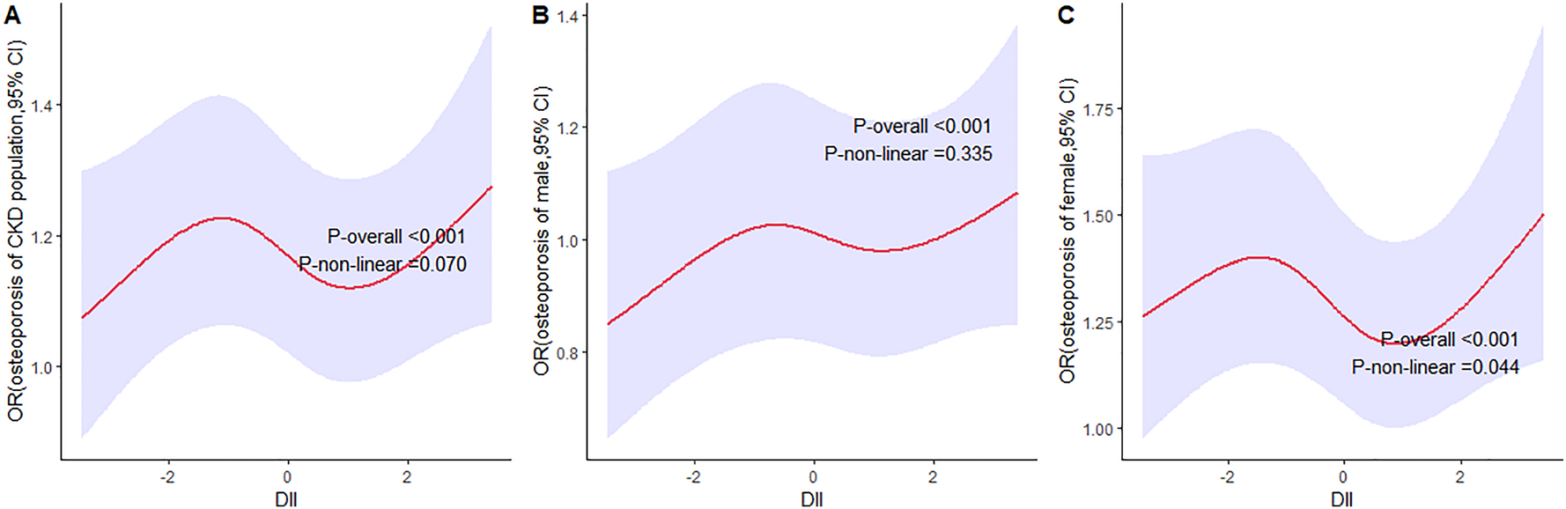
Adjusted ORs of osteoporosis in CKD population according to DII. Graphs show ORs for total CKD population (**A**), male CKD population (**B**), and female CKD population (**C**) adjusted for age, gender, race, BMI group, smoking, steroids use, calcium intake, phosphorus intake, serum calcium, serum phosphorus, total 25-hydroxyvitamin D, albumin, estrogen, anti-osteoporosis drugs, and physical activity. Data were fitted by a logistic regression model using restricted cubic splines. Red solid lines indicate ORs, and purple filled areas indicate 95% CIs.

In order to further evaluate the potential non-linear association between DII score and osteoporosis, we modeled on continuous baseline DII scores using restricted cubic splines (RCSs) in full-adjusted logistic regression models. Three knots (25th, 50th and 75th percentiles) were selected (Fig. 3). The shape of the curves was similar in total CKD population, male CKD population, and female population. As is depicted in Fig. 3c, there was a non-linear relationship between DII score and OR in female population (P_nonlinear_ < 0.05), and the risk of osteoporosis in CKD females with a DII score of around 0.6 to 1.1 was lower than those with a DII score above or below this range. But in the general and male CKD population, the nonlinear relationship was not as significant as in female.

### Association between DII and BMD

Table 3 presents a higher BMD level in Q3 compared with Q4 group at lumbar spine in total CKD population after adjusting for covariates including age, gender, race, BMI group, smoking, steroids use, calcium intake, phosphorus, albumin, estrogen, anti-osteoporosis drugs, and physical activity intake in a linear regression model (*P* < 0.05). The coefficient indicated that subjects in Q4 group had a 3.0% decrease in lumbar spine BMD compared with Q3 group. However, there was no association of BMD and DII at the other two sites, nor in the male or female CKD populations in the full-adjusted model.

**Table 3:** Association of DII and BMD at different body sites.

Models	BMD (g/cm^2^)	DII groups	β[95% CI]		
			All group	Male group	Female group
Model 1	Femoral neck	Q1	− 0.022 (− 0.059, 0.014)	− 0.062 (− 0.121, − 0.004)*	− 0.001 (− 0.045, 0.043)
		Q2	0.000 (− 0.036,0.036)	− 0.009 (− 0.063, 0.045)	− 0.018 (− 0.065, 0.028)
		**Q3**	**Reference**	**Reference**	**Reference**
		Q4	0.020 (− 0.016,0.056)	− 0.006 (− 0.059, 0.048)	0.016 (− 0.031, 0.063)
	Total hip	Q1	− 0.033 (− 0.074, 0.007)	− 0.072 (− 0.132, − 0.012)*	− 0.013 (− 0.062, 0.035)
		Q2	0.009 (− 0.031, 0.050)	− 0.005 (− 0.060, 0.050)	− 0.020 (− 0.072, 0.032)
		**Q3**	**Reference**	**Reference**	**Reference**
		Q4	0.018 (− 0.022, 0.058)	− 0.019 (− 0.074, 0.035)	0.009 (− 0.044, 0.061)
	Lumbar spine	Q1	− 0.038 (− 0.081, 0.005)	− 0.069 (− 0.140, 0.002)	− 0.022 (− 0.072, 0.028)
		Q2	− 0.002 (− 0.045, 0.041)	− 0.022 (− 0.088, 0.043)	− 0.022 (− 0.075, 0.032)
		**Q3**	**Reference**	**Reference**	**Reference**
		Q4	0.005 (− 0.038, 0.048)	− 0.032 (− 0.097, 0.033)	− 0.000 (− 0.054, 0.053)
Model 2	Femoral neck	Q1	− 0.003 (− 0.034, 0.028)	− 0.033 (− 0.086, 0.020)	0.014 (− 0.024, 0.053)
		Q2	0.002 (− 0.029, 0.033)	0.008 (− 0.041, 0.057)	− 0.007 (− 0.048, 0.034)
		**Q3**	**Reference**	**Reference**	**Reference**
		Q4	− 0.008 (− 0.039, 0.023)	− 0.014 (− 0.062, 0.034)	− 0.002 (− 0.044, 0.040)
	Total hip	Q1	− 0.012 (− 0.046, 0.022)	− 0.046 (− 0.102, 0.011)	0.006 (− 0.037, 0.049)
		Q2	0.004 (− 0.030, 0.038)	0.010 (− 0.042, 0.061)	− 0.006 (− 0.052, 0.040)
		**Q3**	**Reference**	**Reference**	**Reference**
		Q4	− 0.017 (− 0.051, 0.017)	− 0.027 (− 0.078, 0.024)	− 0.007 (− 0.054, 0.040)
	Lumbar spine	Q1	− 0.032 (− 0.072, 0.009)	− 0.069 (− 0.141, 0.003)	− 0.014 (− 0.063, 0.034)
		Q2	− 0.014 (− 0.055, 0.026)	− 0.020 (− 0.086, 0.046)	-0.015 (− 0.067, 0.036)
		**Q3**	**Reference**	**Reference**	**Reference**
		Q4	− 0.021 (− 0.062, 0.019)	− 0.031 (− 0.096, 0.034)	− 0.015 (-0.067, 0.038)
Model 3	Femoral neck	Q1	− 0.008 (− 0.039, 0.022)	− 0.020 (− 0.072, 0.031)	0.004 (− 0.035, 0.043)
		Q2	0.002 (− 0.027, 0.032)	0.019 (− 0.027, 0.066)	− 0.009 (− 0.048, 0.030)
		**Q3**	**Reference**	**Reference**	**Reference**
		Q4	− 0.011 (− 0.041, 0.019)	− 0.012 (− 0.060, 0.036)	− 0.005 (− 0.045, 0.036)
	Total hip	Q1	− 0.020 (− 0.051, 0.011)	− 0.025 (− 0.076, 0.026)	− 0.015 (− 0.056, 0.026)
		Q2	0.004 (− 0.027, 0.034)	− 0.025 (− 0.022, 0.071)	− 0.011 (− 0.053,0.030)
		**Q3**	**Reference**	**Reference**	**Reference**
		Q4	− 0.024 (− 0.055, 0.007)	− 0.032 (− 0.079, 0.016)	− 0.011 (− 0.054, 0.031)
	Lumbar spine	Q1	− 0.036 (− 0.076, 0.003)	− 0.042 (− 0.111, 0.026)	− 0.032 (− 0.080, 0.016)
		Q2	− 0.017 (− 0.056, 0.021)	− 0.005 (− 0.068, 0.057)	− 0.025 (− 0.072, 0.023)
		**Q3**	**Reference**	**Reference**	**Reference**
		Q4	− 0.030 (− 0.051,− 0.009)*	− 0.037 (− 0.101, 0.027)	− 0.016 (− 0.065, 0.034)

Model 1: Not adjusted for any other variables.

Model 2: Adjusted for age, gender and race.

Model 3: Adjusted for age, gender, race, BMI group, smoking, steroids use, calcium intake, phosphorus intake, serum calcium, serum phosphorus, total 25-hydroxyvitamin D, albumin, estrogen, anti-osteoporosis drugs, and physical activity.

*β* Partial regression coefficient; *CI* Confidence interval; *BMD* Bone mineral density; *p < 0.05.

**Figure 4:**
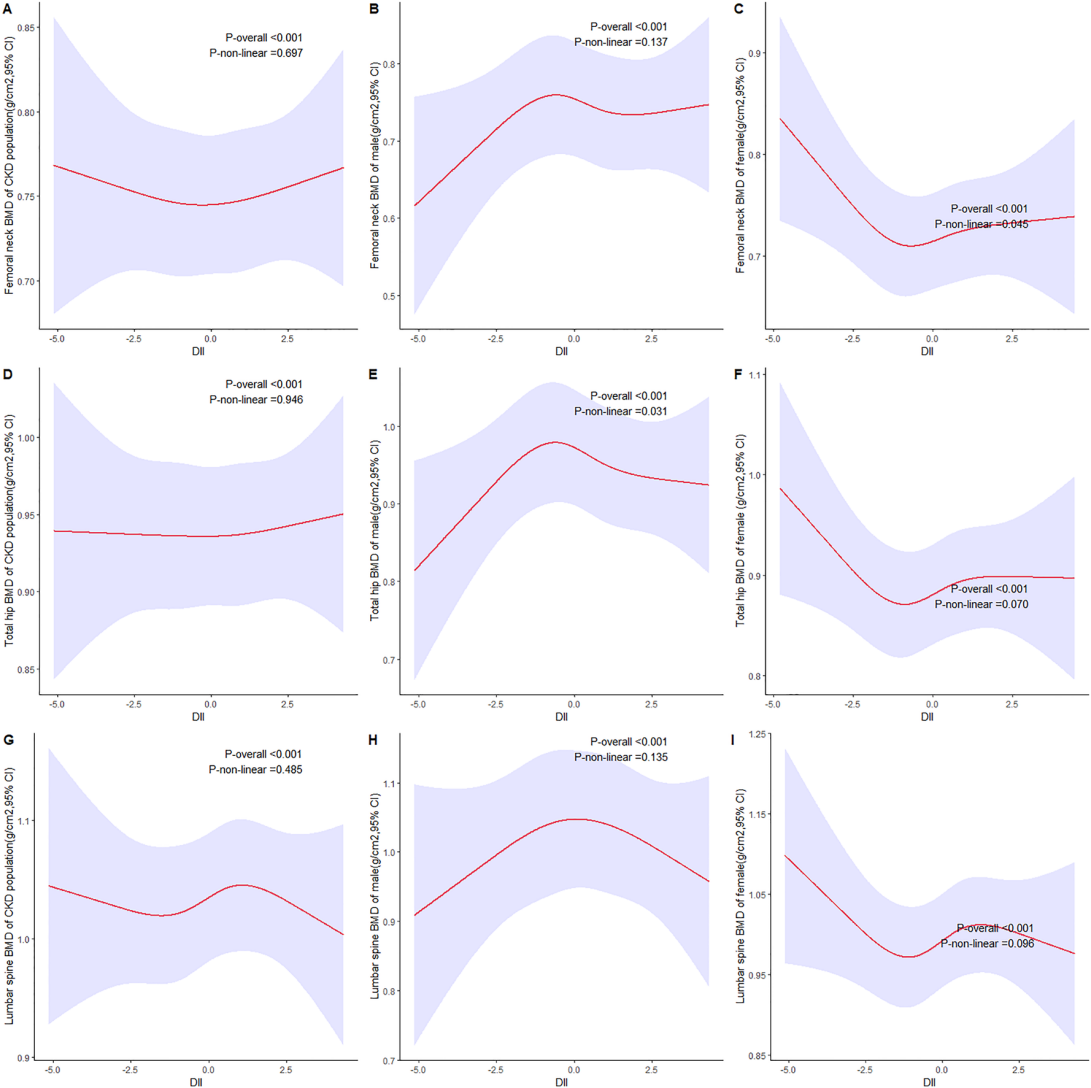
Predicted BMD in CKD population according to DII. Graphs show predicted BMD at three body sites, femoral neck (**A**–**C**), total hip (**D**–**F**), and lumbar spine (**G**–**I**), in total CKD population, male CKD population, and female CKD population respectively. Data were fitted by a linear regression model using restricted cubic splines adjusted for age, gender, race, BMI group, smoking, steroids use, calcium intake, phosphorus intake, serum calcium, serum phosphorus, total 25-hydroxyvitamin D, albumin, estrogen, anti-osteoporosis drugs, and physical activity. Red solid lines indicate predicted BMD, and purple filled areas indicate 95% CIs.

We also adopted RCS method to test our secondary outcomes in full-adjusted linear models at three body sites using continuous baseline DII scores (Fig. 4). The curves varied in different populations this time, especially between male and female. In male, the BMD value at total hip reached the highest when the DII score was around 0, thus forming a reversed U-shape (P_nonlinear_ < 0.05). On the contrary, although there was also a U-shape curve at femoral neck in female, it was not a reversed one (P_nonlinear_ < 0.05). This interesting phenomenon will be discussed in the following section.

## Discussion

In this cross-sectional study based on the NHAHES (2013–2014, 2017–2018) on U.S. population, we included 526 CKD participants identified by their estimated glomerular filtration rates (eGFRs) or albumin-to-creatinine ratios (ACRs). The participants were divided into four groups (Q1, Q2, Q3 and Q4) according to the quantiles of their DII scores (− 5.41 to − 1.22, − 1.22 to − 0.02, − 0.02 to 1.21, and 1.21 to 4.23 respectively). We chose Q3 group as the reference group since the DII values within this group were closest to zero and its osteoporosis incidence rate was the lowest in Table 1 (see the materials and methods section for details). Because gender and age in Table 1 showed significant differences according to DII groups, and the impact of gender or age on osteoporosis has been proved according to previous studies, therefore, in subgroup analysis, we divided the population into four subgroups based on gender and age to explore the effect of DII to different population. In the following RCS method, we also focused on exploring the different impact of gender on outcomes. Our results showed that the risk of osteoporosis was significantly increased in Q4 group compared with Q3 group in adjusted models. In female CKD population, this association was regardless of whether the model was adjusted for other variables. Combined with the RCS plots, our results indicated that DII was positively associated with the risk of osteoporosis when women are on a pro-inflammatory diet. However, this pattern was not that obvious in the male CKD population, probably because the amount of activity of male is generally greater than that of female, and men’s basal metabolic rate is higher^20^. Additionally, hormone changes in sixties, which have the potential to influence various metabolic pathways, differ in male and female and the effects can be very complicated^21^. Therefore, the intake of a pro-inflammatory diet containing more high-quality protein with higher DII might actually conduce to protein anabolism and compensates for the higher protein catabolism than in female. It is worth noting that people with CKD often need more high-quality protein supplement^22,23^. These speculations also apply to the RCS curves in the male population. Men's diet with too low DII, though anti-inflammatory, may suggest insufficient nutritional intake at the same time. Therefore, when DII is less than zero, the BMD at total hip decreases with further reduction of DII. However, subgroup analyses confirmed that the results above were not affected by age, gender, BMI, renal function, urinary protein, serum calcium, serum phosphorus and total 25-hydroxyvitamin D.

There exist researches showing that osteoporosis is connected with inflammatory state of body^7,13,24^. Inflammatory cytokines indirectly mediate bone loss by stimulating the formation and maturation of osteoclasts or by promoting the release of the ligand RANKL^25^. Due to metabolic disorder, CKD patients usually suffer from bone diseases called renal osteodystrophy (ROD), as well as low-grade systemic inflammation and chronic inflammatory status^4,13,26,27^. This results in patients with CKD being exposed to a higher fracture risk because of deteriorated bone quality and quantity^5^. CKD-MBD seems to start very early in CKD patients and some derangements such as phosphate metabolism, adynamic bone disease, FGF23, and klotho secretion, are particularly crucial. Characteristic changes have been seen in serum calcium, phosphorus, active vitamin D, parathyroid hormone, alkaline phosphatase, etc.^10^.

For CKD patients, diet management is crucial. In the past, dietary intervention for renal osteopathy was mainly concentrated on "low phosphorus diet" since high phosphorus is considered as the trigger for a cascade of mineral and bone disorder^6,16,28^. Nowadays, researchers have found that diet is an important factor affecting the inflammatory state of the body. For example, a study found that level of inflammatory factors like TNF-α, IL-1 and IL-2, varied from different dietary pattern^15^. However, few studies focus attention on the inflammatory potential of diets in CKD patients, and our study is the first to explore the relationship between dietary inflammation and osteoporosis in CKD population. Our study results underlined the differences in the effects DII exerted on BMD between male and female might provide innovative information on the underlying sex-specific pathogenetic mechanisms of CKD-MBD.

DII was developed to evaluate the total inflammatory potential of the diet^14^, which have been proved to be highly correlated with inflammatory diseases, such as obesity, diabetes, cardiovascular disease, nonalcoholic fatty liver disease, periodontitis, and CKD^17,29–31^.The calculation of DII score covers all food ingredients that regulate inflammation. As a quantitative indicator, it can reflect the relationship between diet and disease more accurately compared with a single nutrient^32^.

Only a few studies have explored the relationship between DII and bone health, and most of them were conducted in populations of postmenopausal women. A study has been confirmed that there was a negative correlation between DII and BMD in the Middle Eastern population^32^. This study of 160 postmenopausal women aged 50–85 in Iran found that in women with higher DII, BMD of lumbar spine was significantly lower than that of women from the same age group, but no significant correlation was observed in BMD of femoral neck^32^. Orchard et al.^33^ found that a diet of high inflammatory potential was significantly associated with an increased risk of osteoporosis in women, but not in men. Moreover, Zhao et al.^19^ observed a negative correlation between DII and BMD at both total hip and femoral neck sites in female, while the same correlation was observed at lumbar spine in male. These results are partly in accordance with our study in which male and female populations were different. The differences between male and female populations may be attributed to the vital role hormone plays in regulating metabolism and activity of inflammatory cytokines, such as IL-1, IL-6 and TNF-α^24^. This discrepancy might as well be caused by differences in nutrition intake, spinal structure, spinal load and other risk factors between men and women^34^.

This study has some limitations. Firstly, the cross-sectional study only allows us to investigate the relationship rather than causal inference between DII and osteoporosis. Thus, further prospective studies and even clinical trials are needed to confirm this point. Secondly, CKD is defined only by ACR and creatinine measurements so that participants may be incorrectly classified as CKD patients. Thirdly, the DII scores for each subject was calculated using 24-h dietary recall data. However, the process of developing osteoporosis and the change in BMD depends on long-term dietary. Fourthly, after screening CKD patients from general population, the sample size of osteoporosis patients was relatively small to make an accurate conclusion. Lastly, some important variables linked with bone metabolism (e.g., parathormone), as well as some medication information that might affect bone mass^35^, were not included in the study due to a lack of relevant information in the NHANES database. But we tried our best to control the bias. For example, we adjusted serum calcium and phosphorus to decrease the bias, which were deeply related to the effect of PTH on bone metabolism. And in subgroup analysis, we divided whole group into 4 subgroups according to gender and age, to simulate sex hormone changes with age and gender as much as possible. Nevertheless, our research has certain advantages. Good representativeness of random samples from the U.S. NHANES database may result in good extrapolation of the results. Also, we have conducted multiple regression, subgroup analysis, and RCS method from multiple perspectives to exclude possible impact of socio-demographic and other related confounding factors.

In conclusion, a diet with high inflammatory potential was significantly associated with a decrease in lumbar spine BMD and an increase in the risk of osteoporosis in CKD population. Due to the drawbacks of our study and the heterogeneity of the results from different studies, more prospective studies targeted at this subject are expected to verify the role of anti-inflammatory diet in preventing osteoporosis in CKD population in the future.

## Materials and methods

### Study population and data collection

Data in this research were collected from the NHANES on U.S. population across 2 cycles, 2013–2014 and 2017–2018. The NHANES was approved by the National Center for Health Statistics Research Ethics Review Board and received written informed consent from all participants. The inclusion criteria were: (1) individuals with eGFR < 60 ml/min/1.73 m^2^ or ACR > 30 mg/g; (2) individuals aged ≥ 40 years. The exclusion criteria were: (1) individuals with missing data in terms of diet, dual-energy X-ray absorptiometry (DXA) and related covariates; (2) individuals with extreme energy intake (< 500 kcal or > 5000 kcal per day for female and < 500 kcal or > 8000 kcal per day for male)^36–39^. Other indicators collected covered demographics data, examination data, laboratory data, questionnaire and dietary data. Finally, 526 patients were included in our study. This sample size met the estimated sample size (Figure S1). All methods were performed in accordance with the relevant guidelines and regulations. The study was performed in accordance with the Declaration of Helsinki.

### Definitions for CKD and osteoporosis

We used the Modification of Diet in Renal Disease Study Equation to calculate eGFR^40^. CKD was then defined as eGFR < 60 ml/min/1.73 m^2^ or ACR ≥ 30 mg/g^40,41^.

The BMD indexes of lumbar spine, femoral neck and total hip were measured by DXA, and then converted into T scores using the following formula: T score = (BMD respondent−average BMD reference group)/standard deviation (SD) reference group^42^. T ≤  − 2.5 at any one of the three sites mentioned above was defined as osteoporosis^43^.

### Calculation of DII

DII is a scoring system to quantify the inflammatory potential of dietary components proposed by Shivappa et al.^14^, which can be calculated based on the pro-inflammatory and anti-inflammatory properties of 45 different food components to evaluate the inflammation impact of diet^40^. DII was regarded as a major exposure variable in our study and was accessed using 28 nutrients since previous studies had confirmed that DII scores based on 27 or 28 food parameters also ensured good prediction ability^31,44^. The 28 nutrients in this studywere alcohol, β-carotene, caffeine, carbohydrate, cholesterol, energy, total fat, fiber, folic acid, iron, magnesium, monounsaturated fatty acids, polyunsaturated fatty acids, n-3 fatty acids, n-6 fatty acids, protein, saturated fat, selenium, zinc, vitamin A, B1, B2, B3, B6, B12, C, D, and E. The average value of nutrients on the first and second day of the 24-h dietary recall was selected as the nutrient intake level of each subject. Each nutrient parameter was scored according to whether it increased (+ 1), decreased (–1), or had no effect (0) on the inflammatory biomarkers. These scores were weighted based on the study design and were called inflammatory effect scores. By adding each DII score, we can achieve an individual "overall DII score". The detailed calculation process of DII is shown in Fig. 1^14^.

### Potential cofounders

Demographic factors included age, gender, race and income. Race was divided into 5 groups: Mexican American, other Hispanic, non-Hispanic White, non-Hispanic Black, and other races. Income was evaluated by the poverty income ratio (PIR) where PIR < 1 was considered as poor, 1–3 as near-poor, and ≥ 3 as not poor^45^. Behavioral variables consisted of cigarette smoking, alcohol drinking, and bone-affecting drug uses. People who consumed more than 0 g of alcohol in the last 12 months were defined as alcohol consumers and those who smoked more than 100 cigarettes in their lifetime were defined as smokers. Bone-affecting drugs included steroids, estrogen, and anti-osteoporosis medication. Steroids use referred to people who had ever taken prednisone or cortisone daily. Estrogen use encompassed individuals who reported the utilization of any form of estrogen for purposes other than birth control or infertility treatment for a duration over 3 months. Anti-osteoporosis drug use included individuals who had been prescribed medication specifically for the treatment of osteoporosis by a doctor or other health care professional. According to the Physical Activity Guidelines for Americans (2nd edition)^46^, physical activity was defined as having any of the following three conditions: (i) do more than 149 min of moderate physical activity per week; (ii) do more than 74 min of heavy physical activity per week; (iii) do more than 500–1,000 MET-minutes per week. MET is shortened for metabolic equivalent of task. To put it simply, 1 MET represents the energy expended at rest, and a 4 MET activity for 30 min accumulates 120 MET-minutes (2.0 MET-hours) of physical activity. Comorbidities included diabetes, hypertension, cardiovascular diseases, malignant tumors, and strokes. Respondents who answered yes to the following questions were classified as being diagnosed with the corresponding disease: "Have you ever been informed by a health professional or a doctor that you had hypertension/diabetes/cardiovascular disease/malignant tumor/stroke??". BMI was calculated using the formula: body weight (kg)/body height^2^ (m^2^). Calcium and phosphorus intake was extracted from dietary of the past 24 h. Other laboratory indicators were hemoglobin, neutrophil to lymphocyte ratio, albumin, serum calcium, phosphorus level and total 25-hydroxyvitamin D. Total 25-hydroxyvitamin D was defined as sum of 25-hydroxyvitamin D2 and 25-hydroxyvitamin D3, excluding epi-25-hydroxyvitamin D3. The procedures and methods for collecting and analyzing urine and blood samples in the NHANES have been comprehensively described at https://wwwn.cdc.gov/nchs/nhanes/Default.aspx. Notably, as the computation of the DII value incorporated energy factors, the inclusion of the energy variable as a covariate within the model was not necessary^47–49^.

### Outcome variables

The primary outcome was osteoporosis. The secondary outcomes were BMD values from 3 body sites: total hip, femoral neck and lumbar spine.

### Statistical analysis

Characteristics of participants in this study were descriptively analyzed for all individuals and within four DII subgroups divided by DII quantiles. Normality of distribution was tested using *Kolmogorov–Smirnov's* test. For normally distributed data, quantitative variables were presented as means ± SDs and were compared using a *t*-test. For non-normally distributed variables, quantitative variables were presented as medians and interquartile ranges (IQRs), and were compared using *Mann–Whitney* test. Categorical variables were expressed as percentages or frequencies and were compared by *chi-squared* test. Multivariable logistic regression models were used to examine the associations between DII and osteoporosis, while multivariable linear regression models were used to evaluate the association between DII and BMD. In models, DII was categorized into four subgroups by the quantiles of DII distribution, with quantile 3 serving as the reference group. Three models were developed: model 1 was not adjusted for any other variables; model 2 was adjusted for age, gender, and race; model 3 was additionally adjusted for BMI group, smoking, steroids use, calcium intake, phosphorus intake, serum calcium, serum phosphorus, total 25-hydroxyvitamin D, albumin, estrogen, anti-osteoporosis drugs, and physical activity further. The adjusted confounding variables in these models were determined based on variables with *P* values less than 0.05 in Table 1 and variables identified from previous research which has direct or indirect potential influence on osteoporosis^19,50,51^. All models were fitted separately for the total CKD population, male CKD population, and female CKD populations. The range of DII within the Q3 group spanned across zero, which indicates a dietary pattern neither promoting nor inhibiting inflammation. Furthermore, according to Table 1, the Q3 group appeared to exhibit the lowest incidence rate of osteoporosis. Hence, the Q3 group was considered as the reference group to investigate the association between DII and the incidence of osteoporosis. Additionally, subgroup analyses stratified according to age, gender, renal function, ACR and BMI were performed. RCS method was also used to explore the possible nonlinear relationship between DII and osteoporosis risk. Survey sampling weights were considered throughout all analyses. All statistical analyses were conducted with SPSS 25.0 (IBM, Armonk, NY, USA) and R statistical software version 4.1.2 (R Foundation), and *P *values < 0.05 were considered statistically significant.

### Supplementary Information

Supplementary Information.

## Data Availability

The data supporting the findings of this study are available from the corresponding author upon request.

## Abbreviations

ACR: Albumin-to-creatinine ratio
aOR: Adjusted odds ratio
BMD: Bone mineral density
BMI: Body mass index
CDC: Center for Disease Control and Prevention
CI: Confident interval
CKD: Chronic kidney disease
CKD-MBD: Chronic kidney disease-mineral bone disorder
DII: Dietary inflammatory index
DXA: Dual-energy X-ray absorptiometry
eGFR: Estimated glomerular filtration rate
FGF23: Fibroblast growth factor 23
IQR: Interquartile range
NHANES: National Health and Nutrition Examination Survey
NLR: Neutrophil to lymphocyte ratio
OPG: Osteoprotegerin
OR: Odds ratio
PIR: Poverty income ratio
PTH: Parathyroid hormone
RANKL: Receptor activator of nuclear factor kappa-B ligand
RCS: Restricted cubic spline
ROD: Renal osteodystrophy
SD: Standard deviation
Th17: T-helper 17
