# Supplementary material for: The relationship between dietary inflammatory index and osteoporosis among chronic kidney disease population
Source: Sci Rep. 2023 Dec 18;13:22867. doi: 10.1038/s41598-023-49824-5 (PMC10739725; doi:10.1038/s41598-023-49824-5)
Supplement: Supplementary file 1 — Supplementary Information. [file 41598_2023_49824_MOESM1_ESM.docx]

**Table S1 Comparison of each component of DII scores between DII quartile groups**

| **Variables** | **DII** | | | | | ***P*** |
| --- | --- | --- | --- | --- | --- | --- |
|  | **Overall**  **N=526** | **Q1**  **N=131** | **Q2**  **N=132** | **Q3**  **N=131** | **Q4**  **N=132** |  |
| Alcohol | 0[0,0] | 0[0,0]^a^ | 0[0,0]^b^ | 0[0,0]^c^ | 0[0,14]^abc^ | <0.001** |
| β-carotene | 812[263,2660] | 2760 [1130, 6015]^abc^ | 1112 [443, 3010]^ade^ | 520 [213, 1696]^bd^ | 253 [85, 619]c^ce^ | <0.001** |
| Caffeine | 0.1[0.02,0.20] | 0.09[[0.01, 0.18]^a^ | 0.10 [0.01, 0.19]^b^ | 0.10 [0.04, 0.18] | 0.12 [0.04, 0.23]^ab^ | 0.006** |
| Carbohydrate | 208[146,292] | 181 [142, 245]^a^ | 210 [145, 292]^b^ | 223 [148, 291] | 237 [152, 335]^ab^ | <0.001** |
| Cholesterol | 225[124,382] | 201 [110, 348] | 220 [126, 403] | 234 [123, 381] | 252 [135, 404] | 0.407 |
| Energy | 1759[1291,2450] | 1404 [1074, 1919]^ab^ | 1711 [1356, 2445]^c^ | 1801 [1351, 2367]^ad^ | 2021 [1494, 2823]^bcd^ | <0.001** |
| Total fat | 66[44,96] | 47 [36, 74]^abc^ | 67 [46, 91]^ad^ | 72 [48, 99]^b^ | 80 [51, 116]^cd^ | <0.001** |
| Fiber | 13.3[8.9,20.8] | 17.0 [11.8, 27.5]^ab^ | 15.7 [10.7, 24.8]^cd^ | 11.9 [7.9, 19.7]^ac^ | 9.8 [5.5, 14.4]^bd^ | <0.001** |
| Folic acid | 111[52,209] | 116 [36,223] | 119 [55, 212] | 107[[65, 181] | 107 [50, 203] | 0.152 |
| Iron | 11.4[7.9,16.6] | 12.2 [8.5, 19.3]^a^ | 12.1[8.2, 16.1] | 11.1 [7.8, 16.7] | 9.8 [6.7, 14.7]^a^ | 0.019* |
| Magnesium | 246[181,346] | 282 [221, 393]^ab^ | 259 [198, 371]^c^ | 233 [173, 309]^a^ | 207 [142, 303]^bc^ | <0.001** |
| Monounsaturated fatty acids | 22[15,33] | 16 [11, 25]^abc^ | 22 [14, 33]^a^ | 24 [16, 34]^b^ | 25 [18, 39]^c^ | <0.001** |
| Polyunsaturated fatty acids | 14.8[9.5,22.5] | 12.7 [8.8, 18.8] | 16.0 [9.6, 24.1] | 16.4 [10.4, 23.0] | 14.1 [9.0, 23.4] | 0.053 |
| n-3 fatty acids | 1.45[0.89,2.27] | 1.41 [0.87, 2.09] | 1.56 [0.92, 2.45] | 1.46 [0.90, 2.22] | 1.36 [0.78, 2.21] | 0.773 |
| n-6 fatty acids | 13.2[8.4,20.1] | 11.1 [7.7, 16.7]^a^ | 15.0 [8.5, 21.7] | 14.6 [9.4, 20.9]^a^ | 12.7 [8.3, 21.0] | 0.026* |
| Protein | 66[49,92] | 63 [49, 90] | 71 [50, 95] | 64 [48, 93] | 63 [44, 89] | 0.463 |
| Saturated fat | 21[12,31] | 13 [9, 21]^abc^ | 22 [12, 29]^ad^ | 22 [14, 33]^be^ | 27 [17, 39]^cde^ | <0.001** |
| Selenium | 91[66,129] | 87 [64, 128] | 98 [70, 133] | 88 [65, 127] | 91 [65, 128] | 0.468 |
| Zinc | 8.8[5.9,12.8] | 9.1 [6.0, 12.9] | 9.3 [6.6, 14.5] | 8.0 [5.5, 11.7] | 8.5 [4.9, 11.4] | 0.151 |
| Vitamin A | 442[241,751] | 666 [421, 1189]^abc^ | 468 [249, 774]^ad^ | 389 [203, 628]^b^ | 307 [150, 500]^cd^ | <0.001** |
| Vitamin B1 | 1.25[0.89,1.91] | 1.36 [0.89, 2.03] | 1.33 [0.94, 1.93] | 1.23 [0.91, 1.90] | 1.20 [0.73, 1.62] | 0.184 |
| Vitamin B2 | 1.6[1.28,2.3] | 1.6 [1.1, 2.5] | 1.7 [1.3, 2.2] | 1.6 [1.2, 2.2] | 1.4 [1.0, 2.2] | 0.376 |
| Vitamin B3 | 20 [13,28] | 19 [13, 32] | 21 [14, 28] | 21 [13, 27] | 19 [12, 25] | 0.151 |
| Vitamin B6 | 1.5[1.1,2.3] | 1.9 [1.3, 2.7]^ab^ | 1.6 [1.1, 2.4]^c^ | 1.5 [0.9, 2.1]^a^ | 1.2 [0.8, 1.8]^bc^ | <0.001** |
| Vitamin B12 | 3.2[1.7,5.5] | 3.6 [1.6, 6.2]^a^ | 3.3 [1.6, 5.5] | 3.2 [1.7, 5.1] | 2.9 [1.7, 5.3]^a^ | 0.014* |
| Vitamin C | 49[18,111] | 97 [49, 160]^ab^ | 57[28, 116]^c^ | 37[16, 96]^a^ | 17 [7, 52]^bc^ | <0.001** |
| Vitamin D | 2.7[1.0,5.1] | 3.6 [1.4,6.4]^ab^ | 2.9 [1.4, 6.0]^c^ | 2.4 [0.9, 4.7]^a^ | 1.8 [0.8, 4.1]^bc^ | <0.001** |
| Vitamin E | 6.6[4.4,10.5] | 7.1 [4.9, 12.2]^ab^ | 6.9 [4.8, 11.2] | 6.6 [4.5, 9.7]^a^ | 5.3 [3.2, 8.9]^b^ | 0.001** |

a,b,c,d,e: statistically significance between 2 groups using the *Bonferroni* method.


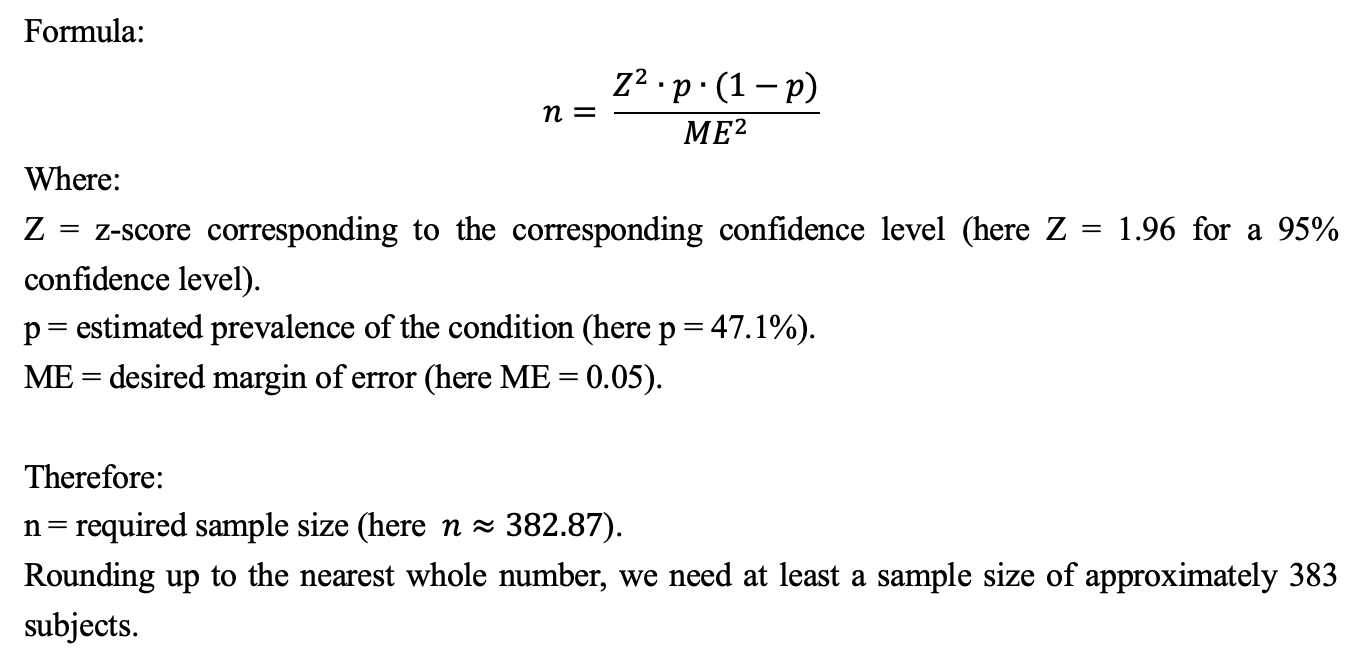


**Figure S1| Sample size calculation method and results for this cross-sectional study.** Here p value referred to the estimated prevalence of osteoporosis in CKD population (Kinsella S, Chavrimootoo S, Molloy MG, Eustace JA. Moderate chronic kidney disease in women is associated with fracture occurrence independently of osteoporosis. Nephron Clin Pract. 2010;116(3):c256-62. doi: 10.1159/000317207. Epub 2010 Jul 2. PMID: 20606487.).
